# Risk factors for interstitial lung disease: a 9-year Nationwide population-based study

**DOI:** 10.1186/s12890-018-0660-2

**Published:** 2018-06-04

**Authors:** Won-Il Choi, Sonila Dauti, Hyun Jung Kim, Sun Hyo Park, Jae Seok Park, Choong Won Lee

**Affiliations:** 1 Department of Internal Medicine, Keimyung University Dongsan Hospital, Daegu, 41931 Republic of Korea; 2 Department of Allergology, Hospital Serive of Kavaje, Kavaje, Albania; 3 Department of Occupational & Environmental Medicine, Sungso Hospital, Andong, Republic of Korea

**Keywords:** Interstitial lung disease, Epidemiology, Risk factor

## Abstract

**Background:**

Understanding the risk factors that are associated with the development of interstitial lung disease might have an important role in understanding the pathogenetic mechanism of interstitial lung disease as well as prevention. We aimed to determine independent risk factors of interstitial lung disease development.

**Methods:**

This was a retrospective cohort study with nationwide population-based 9-year longitudinal data. We selected subjects who were aged > 40 years at cohort entry and with a self-reported history of cigarette smoking. Cases were selected based on International Classification of Diseases codes. A cohort of 312,519 subjects were followed until December 2013. We used Cox regression analysis to calculate the hazard ratios (HRs) for interstitial lung disease development.

**Results:**

Interstitial lung disease developed in 1972 of the 312,519 subjects during the 9-year period. Smoking (HR: 1.2; 95% confidence interval [CI]: 1.1–1.4), hepatitis C (HR: 1.6; 95% CI: 1.1–2.3), history of tuberculosis (HR: 1.5; 95% CI: 1.1–1.9), history of pneumonia (HR: 1.6; 95% CI: 1.3–2.0), and chronic obstructive pulmonary disease (HR: 1.8; 95% CI: 1.6–2.1), men (HR: 1.9; 95% CI: 1.7–2.1) were significantly associated with the development of interstitial lung disease. The risk of interstitial lung disease development increases with age, and the risk was 6.9 times higher (95% CI: 5.9–8.0) in those aged over 70 than in their forties.

**Conclusions:**

Smoking, hepatitis C, history of tuberculosis, history of pneumonia, chronic obstructive pulmonary disease, male sex, and older age were significantly associated with interstitial lung disease development.

## Background

If bilateral reticular or reticulonodular opacities are found on chest radiography, interstitial lung disease (ILD) is suspected. ILD is not an uncommon disease [1]. In 2012, prevalence estimates of ILD with fibrosis ranged from 42.7–63 per 100.000 population in USA, and 1.25–23.4 per 100.000 population in Europe [2].

Patients with ILD are often asymptomatic until the lesion progresses significantly. Interstitial pulmonary abnormality (ILA), which is considered to be an early lesion of interstitial lung disease, has a higher mortality rate than patients without ILA [3] as well as ILD [4]. Furthermore, in patients with ILD, a study using health insurance claim data found that lung cancer incidence is higher than chronic obstructive pulmonary disease (COPD) [5]. ILD could have significant impact on health.

Three different population-based studies determined that cigarette smoking is a risk factor for lung parenchymal as well as interstitial abnormalities in addition to airway abnormalities [6–8]. However, previous studies examined risk factors from a limited sample size, and most of them used case-control methodology and did not consider many of the potential confounding variables as risk factors for ILD.

Determining the risk factors that are associated with the development of ILD might have an important role in understanding the pathogenetic mechanism of ILD, early diagnosis, adequate treatment and prevention.

The purpose of this study was to identify the independent risk factors for the development of ILD in a 9-year follow-up longitudinal population-based study.

## Methods

### Database

The National Health Insurance Service (NHI) covers more than 99% of all Korean residents and includes all health claim data, including diagnostic codes, procedures, prescription drugs, patient personal information, and hospital information. There is one health insurance system with a unique resident registration number for each citizen; therefore, duplication of subjects can be avoided. This study used data from the National Health Insurance Service-National Sample Cohort(NHIS-NSC) 2002–2013 [9], which was released by the KNHIS in 2015. It includes all medical claims filed from January 2002 to December 2013 for 1,099,094 nationally representative randomly selected subjects, accounting for approximately 2.2% of the entire population in the KNHIS in 2002. The data were produced by the KNHIS using a systematic sampling method to generate a representative sample of all 46,605,433 Korean residents in 2002. The cohort population underwent biennial medical evaluations through the NHI Corporation between January 1, 2002, and December 31, 2013.

### Study population

This was a nationwide population-based 9-year longitudinal study. We included subjects with a self-reported smoking history, who were ≥ 40 years old, and with co-morbidities diagnosed before the index date. Health examination data confirmed self-reported cigarette smoking history. The health examination data every 2 years was linked with the cohort data.

We did not include patients with ILD from 2002 to 2004 to exclude preexisting cases of ILD during the two medical evaluations and 1 year of follow-up. We also excluded patients with ILD who did not visit the clinic 30 days after the index date. The final sample included 312,519 subjects. Then, we identified patients with newly diagnosed ILD between January 2005 and December 2013. Among the 312,519 subjects, the control group was selected by excluding those who had ILD between 2005 and 2013.

### Definition of interstitial lung disease

Cases of ILD were selected based on International Classification of Disease-10 (ICD-10) code J84 for other interstitial lung diseases, excluding drug-induced interstitial lung disorders, interstitial emphysema, and lung diseases caused by external agents. Connective tissue disease-associated ILD, hypersensitivity pneumonitis, and sarcoidosis were excluded.

### Comorbidities

Comorbidities diagnosed before the index date that could be associated with an increased risk of lung fibrosis were identified using ICD-10, including COPD, hepatitis C, gastroesophageal reflux disorder (GERD), and diabetes [10–13].

### Statistical analysis

Baseline characteristics (including age, sex, and comorbidities) for cases and controls are summarized using descriptive statistics such as proportion. A chi-squared test was used to compare frequencies of risk factors between ILD and the control group. Cox proportional hazards regression models were used to evaluate the risk factors for ILD and analyze the associations between ILD and different variables and comorbidities. The final multivariate models included age, sex, smoking status (former or current smoker vs. never smoker), household income, and comorbidities such as hepatitis C, herpes, tuberculosis, pneumonia, GERD, COPD, diabetes, and hepatitis B. Risk factor models for ILD were selected according to sex and smoking as sensitivity analysis. Model selection method was forward stepwise procedure using likelihood ratio test with *p*-value < 0.05 as entry criterion, and p-value ≥0.10 as removal criterion.

A *P* value < 0.05 considered to be statistically significant. All statistical analyses were performed using SAS V.9.2 (SAS Institute, Cary, North Carolina, USA).

## Results

### Incidence and baseline characteristics

**Fig. 1:**
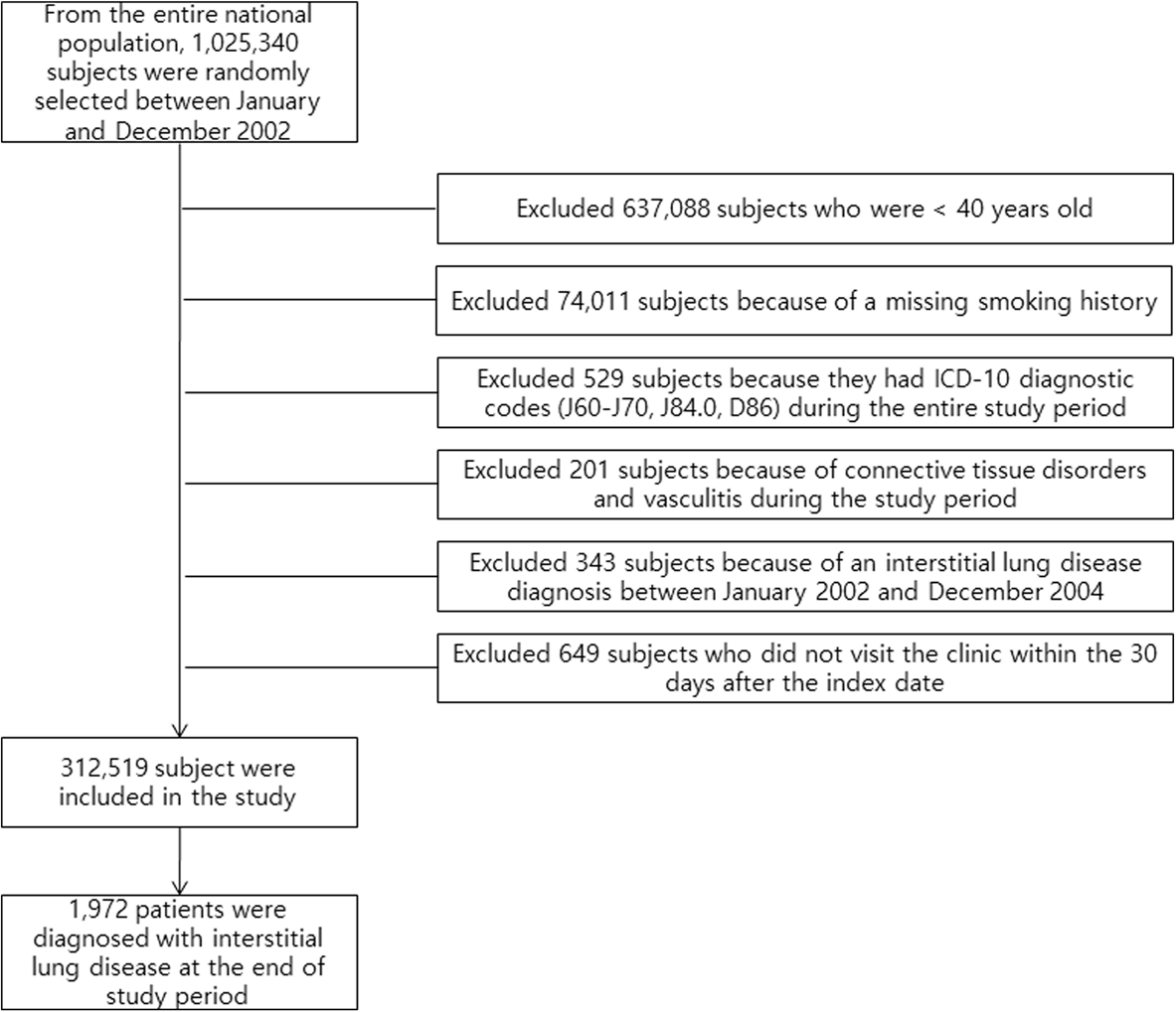
Flow chart of the study for selection of patients with interstitial lung disease (ILD). ICD: International Classification of Disease

The final sample included 312,519 subjects, of which 1972 developed ILD during the 9-year study period (Fig. 1). ILD incidence was 70.1 cases per 100,000 person-year.

All subjects were tracked by December 31, 2013, Follow up duration was median 65.6 months (interquartile range: 35.5, 89.0) in the ILD group and median 107.5 months (interquartile range; 100.2, 109.5) in the non-ILD group.

The ILD group had a higher percentage of men than the control. Compared with the control, subjects with ILD were older, and more likely to be smokers. The ILD group was more likely to have comorbidities such as respiratory diseases (tuberculosis and pneumonia), diabetes, chronic renal failure, malignancy, GERD, hepatitis C, and COPD than the control (Table 1).

**Table 1:** Baseline characteristics of patients with interstitial lung disease (*n* = 1972) and controls (*n* = 310,547)

Variable	Interstitial lung disease No. (%)		Control No. (%)		*P* value
Sex					
Male	1265	(64.1)	151,124	(48.7)	< 0.001
Female	707	(35.9)	159,423	(51.3)	
Age group (years)					
40–49	354	(18.0)	136,576	(44.0)	< 0.001
50–59	474	(24.0)	89,952	(29.0)	
60–69	662	(33.6)	57,189	(18.4)	
≥ 70	482	(24.4)	26,830	(8.6)	
Baseline comorbidity					
Malignancy	357	(18.1)	43,007	(13.8)	< 0.001
Diabetes	479	(24.3)	48,507	(15.6)	< 0.001
Chronic renal failure	27	(1.4)	2187	(0.7)	< 0.001
COPD^a^	242	(12.3)	11,204	(3.6)	< 0.001
Past history					
Herpes (B00, B02)	65	(3.3)	9997	(3.2)	0.85
Tuberculosis (A15, A16, B90)	102	(5.2)	5467	(1.8)	< 0.001
Pneumonia (J12-J18)	98	(5.0)	6709	(2.2)	< 0.001
Hepatitis C (B18.2)	28	(1.4)	2068	(0.7)	< 0.001
Hepatitis B (B18.0, B18.1)	66	(3.3)	9859	(3.2)	0.66
GERD^b^ (K21)	227	(11.5)	26,643	(8.6)	< 0.001
Smoker (ex-smoker or current smoker)	748	(37.9)	93,573	(30.1)	< 0.001
Household income (Quartiles), %					
81–100	870	(44.1)	131,363	(42.3)	0.11
41–80	635	(32.2)	107,310	(34.6)	
11–40	437	(22.2)	66,372	(21.4)	
0–10	30	(1.5)	5502	(1.8)	

^a^*COPD* chronic obstructive pulmonary disease, ^b^ *GERD* gastroesophageal reflux disorder

### Risk factors for developing ILD

Based on a multivariate Cox regression analysis of all variables (Table 2), smoking was significantly associated with the development of ILD (HR: 1.2; 95% CI: 1.1–1.4).

**Table 2:** Univariate and multivariate Cox regression analyses for development of interstitial lung disease during the 9-year follow-up period

Risk factor	Univariate analysis		Multivariate analysis	
	HR (95% CI)	*P* value	HR (95% CI)	*P* value
Men (reference: women)	1.9 (1.7–2.1)	< 0.001	1.9 (1.7–2.1)	< 0.001
Age group (years) (reference: 40–49)				
50–59	1.9 (1.7–2.2)	< 0.001	1.9 (1.7–2.2)	< 0.001
60–69	4.3 (3.8–4.9)	< 0.001	4.1 (3.6–4.7)	< 0.001
≥70	7.1 (6.2–8.2)	< 0.001	6.9 (5.9–8.0)	< 0.001
Diabetes (reference: no)	1.6 (1.5–1.8)	< 0.001	1.1 (0.9–1.2)	0.06
COPD (reference: no)	3.7 (3.2–4.2)	< 0.001	1.8 (1.6–2.1)	< 0.001
GERD (reference: no)	1.3 (1.1–1.5)	< 0.001	1.0 (0.9–1.3)	0.31
History of herpes (reference: no)	0.97 (0.7–1.2)	0.85	0.9 (0.7–1.2)	0.92
History of tuberculosis (reference: no)	3.0 (2.4–3.7)	< 0.001	1.5 (1.1–1.9)	0.003
History of pneumonia (reference: no)	2.2 (1.8–2.7)	< 0.001	1.6 (1.3–2.0)	< 0.001
Hepatitis C (reference: no)	2.1 (1.4–3.0)	< 0.001	1.6 (1.1–2.3)	0.01
Hepatitis B (reference: no)	1.0 (0.8–1.3)	0.84	0.8 (0.7–1.1)	0.39
Smoker (reference: never smoker)	1.4 (1.3–1.6)	< 0.001	1.2 (1.1–1.4)	< 0.001
Household income, % (reference: 81–100**)**				
41–80	0.8 (0.8–0.9)	0.04	0.9 (0.8–1.0)	0.35
11–40	1.0 (0.9–1.1)	0.85	0.9 (0.8–1.1)	0.87
0–10	1.1 (0.8–1.6)	0.39	1.1 (0.7–1.5)	0.59

*HR* hazard ratio, *CI* confidence interval, *COPD* chronic obstructive pulmonary disease, *GERD* gastroesophageal reflux disorder

Co-morbidities such as hepatitis C (HR: 1.6; 95% CI: 1.1–2.3), history of tuberculosis (HR: 1.5; 95% CI: 1.1–1.9), history of pneumonia (HR: 1.6; 95% CI: 1.3–2.0), and COPD (HR: 1.8; 95% CI: 1.6–2.1) were significantly associated with the development of ILD. Men were almost twice as likely to associated with the development of ILD as women.

The risk of ILD development was 1.9 times in the 50s, 4.1 times in the 60s, and 6.9 times in the 70s, which increased sharply with age. In multivariate analysis, age was found to be most associated to ILD development showing dose response. All the risk factors included in the model showed relatively narrow confidence intervals.

There were no variables that changed the direction of the hazard ratio in the univariate and multivariate analyses except hepatitis B, which was statistically not significant. The hazard ratios of COPD and history of tuberculosis were reduced by almost half after multivariate analysis. History of pneumonia, hepatitis C and diabetes showed also reduced hazard ratios in multivariate analysis. Smoking was associated with the occurrence of ILD, but its magnitude was relatively small compared to other variables.

Multivariate analysis stratified by smoking showed same direction and similar magnitude of hazard ratios in all variables. However, hazard ratios stratified by sex were somewhat different in several variables. As a result of stratified analysis on the basis of gender, the hazard ratios of COPD according to man and woman were similar 1.8 and 1.9, respectively. In the case of GERD, multivariate analysis of male subjects showed that GERD was not a significant risk factor, but the hazard ratio for women was 1.3, which was consistent with the 1.3 observed in the univariate analysis (Table 3). GERD, Hepatitis C are significant risk factors in females, in contrast malignancy and diabetes are significant risk factors in males.

**Table 3:** Multivariate Cox regression analyses with forward stepwise variable selection method for development of interstitial lung disease during the 9-year follow-up period

Risk factor	Males		Females	
	HR (95% CI)	*P* value	HR (95% CI)	*P* value
Age group (years) (reference: 40–49)				
50–59	2.3 (1.9–2.7)	< 0.001	1.4 (1.1–1.7)	0.004
60–69	4.6 (3.9–5.5)	< 0.001	3.3 (2.6–4.1)	< 0.001
≥70	8.1 (6.7–9.7)	< 0.001	5.4 (4.3–6.7)	< 0.001
Malignancy	1.1 (1.0–1.3)	0.045	1.0 (0.8–1.2)	0.68
Diabetes (reference: no)	1.2 (1.0–1.3)	0.006	0.9 (0.7–1.1)	0.38
COPD (reference: no)	1.8 (1.5–2.2)	< 0.001	1.9 (1.5–2.4)	< 0.001
History of tuberculosis (reference: no)	1.5 (1.2–2.0)	< 0.001	1.8 (1.2–2.6)	0.001
History of pneumonia (reference: no)	1.5 (1.1–2.0)	0.002	1.7 (1.2–2.3)	0.001
Hepatitis C (reference: no)	1.3 (0.8–2.1)	0.26	2.1 (1.1–3.8)	0.014
GERD (reference: no)	1.0 (0.9–1.3)	0.38	1.3 (1.0–1.6)	0.013
Smoker (reference: never smoker)	1.2 (1.1–1.4)	< 0.001	1.5 (1.1–2.0)	0.005

*HR* hazard ratio, *CI* confidence interval, *COPD* chronic obstructive pulmonary disease, *GERD* gastroesophageal reflux disorder

## Discussion

In the present study, the development of ILD was associated with older age, male sex, cigarette smoking, hepatitis C, history of tuberculosis, history of pneumonia, and COPD.

Smokers were at greater risk of developing ILD than non-smokers (HR: 1.2). Similarly, a previous study showed that smoking might contribute to the development of ILD by fibrosis (Odds Ratio: 1.6) [14]. The findings of three different studies also support a strong association between ILA and exposure to tobacco smoke and smoking status [6–8]. In this study, the risk of developing ILD was 1.4 in smokers compared to non-smokers in the univariate analysis, but decreased 1.2-fold in multivariate analysis. The lowest risk among the variables associated with ILD occurrence is low, indicating that smoking is less involved in ILD development.

COPD was associated with the development of ILD in the present study. A 2012 review of the pathogenesis of IPF and COPD showed similarities between the basic pathogenic mechanisms involved in the development of either emphysema or fibrosis [13]. Coexisting pulmonary fibrosis and emphysema is now a distinct entity [15], and studies show that the pathologic changes associated with these coexisting entities are mostly found in smokers [16–19]. In this study, the risk of developing ILD in COPD was 3.7 in univariate analysis but decreased 1.8-fold in multivariate analysis. This is presumably due to the control of cigarette smoking variables.

Hepatitis C was another risk factor associated with the development of ILD in the present study. There have been conflicting results regarding the prevalence of anti-hepatitis C virus (HCV) antibody in patients with IPF [10, 20, 21]. However, a 2008 study with HCV-infected patients and HBV-infected controls showed that ILD with fibrosis developed at a significantly greater rate in the HCV group than in the HBV group [22]. In 2002, Idilman et al. reported an increased bronchoalveolar lavage neutrophil count in individuals with hepatitis C, suggesting an inflammatory reaction in the lungs leading to fibrotic changes [23]. The findings of these studies suggest that systemic factors stimulating fibrosis, such as HCV infection, may affect the development of lung fibrosis.

Local factors such as history of pneumonia or tuberculosis may be associated with lung fibrosis. Nonresolving pneumonia may result in organizing pneumonia commonly in bacterial infections [24]. Pneumonia due to mycoplasma and Legionnaires’ disease has been mostly implicated with development of pulmonary fibrosis [25, 26]. However, the occurrence of organization in cases of pneumonia is more common than expected. In 1952, Auerbach et al. studied the material from 307 necropsies and found organization in 38 cases [27]. In 1989, Shachor et al. found that the incidence of tuberculosis in subjects with ILD was 4.5 times higher than that of the general population [28]. Dheda et al. studied lung remodeling and fibrosis associated with lung injury from tuberculosis infection. Lung remodeling can result in extensive fibrosis and may be interstitial [29]. Therefore, ILD may develop due to tuberculosis or other lung infections.

GERD is a well-known risk factor for IPF [11, 30–32]. However, our data did not show a significant association between GERD and ILD. In patients with esophagitis, the associated odds ratio of pulmonary fibrosis was 1.3–1.6 [30, 33], but there was no significant association between reflux esophagitis and pulmonary fibrosis in this study (Table 2). However, GERD were observed only in women as a significant risk factor for the development of ILD (Table 3). In the present study, ICD-10 code defined a wider range of ILD. Therefore, in this study, it is estimated that mild cases of pulmonary fibrosis are more involved than previous studies, and that there was no significant association between reflux esophagitis and ILD in men.

Diabetes is also prevalent with IPF [12, 33]. Although diabetes was an important risk factor for developing ILD in the univariate analysis, the significance reduced to borderline in the multivariate analysis (HR 1.1, *P* = 0.06).

Our study shows that men and older individuals have a higher risk of developing ILD. Two other studies conducted with subjects with interstitial abnormalities found that they were significantly older [7, 8]. Several studies have shown that the incidence of ILD with fibrosis is higher in men and increases with advanced age [34–37]. In our study, the HR for developing ILD in those aged ≥70 years was almost 7 times higher than for those aged 40–49 years. We suggest that ILD would be a result of the aging process.

We found reduction of risk estimates from the univariate to the multivariate analysis of some variables such as COPD and GERD. We did sensitivity analysis based on gender. Both males and females have similar risk estimates for ILD development in relation to COPD. However, GERD is significant risk factor in females but not in males.

ILD is known as non-homogeneous diseases. Although assessing risk factors for developing specific forms of ILD would be ideal, it might be difficult performing such kinds of study because surgical lung biopsy was rarely performed. In addition, assessing risk factors for the ILD development, the control group should be a general population. Therefore, we assess risk factors for developing ILD by population-based cohort database, though it does not provide information on specific forms of ILD.

The incidence of ILD was 70.1 cases per 100,000 per year, which is higher than previously reported [38]. However, the present incidence was calculated for individuals > 40 years old.

### Limitations of the present study

The most important limitation of the present study is that the diagnoses of ILD and other comorbidities were defined based on ICD codes, which may be inaccurate compared to the diagnoses obtained from a medical chart. Underreporting of asymptomatic ILD or misclassification was also possible.

The validity of the medical insurance claims data for ILD has not been determined in Korea. This database consists of random samples of national insurance claim data without identification numbers. Therefore, it was impossible to validate individual cases through a chart review.

We may have underestimated ILD incidence because of inaccurate ILD data. However, previous studies of ILD incidence using data from the Health Insurance Review and Assessment Service of Korea [38] reported similar results to those of previous studies [35, 36, 39]. The incidence rate of ILD was reportedly 48.5 per 100,000 person-years in Korea based on all claims data from 2008 to 2012 [38]. In the present study, the incidence rate was 70.1 per 100,000 patients from 2005 to 2013, which appears reasonable because we excluded subjects aged < 40 years.

The present study did not take into consideration of relevant risk factor such as occupational and environmental exposure. However, annual income could be a weak proxy of the occupational exposure [40].

The present study may be affected by selection bias because the controls were identified based on medical claims. Thus, the controls were more likely to have comorbidities than controls selected from the general population. Although we excluded patients with a diagnosis of ILD between January 2002 and December 2004 to exclude pre-existing ILD diagnosed before the first year of our study (2005), patients with ILD could be miscounted as new cases if the patient did not require medical care between 2002 and 2004, which may have caused inaccuracies. In addition, other risk factors, such as pulmonary function and high-resolution computed tomography findings for ILD, could not be evaluated because of the NHIS-NSC 2002–2013 primarily included medical claims.

## Conclusions

The population is aging, and the incidence of ILD increases with advancing age. Aged men with the history of previous lung infection or COPD could be a clinical marker of suspect ILD. The demonstration of the association of these clinical attributes with ILD may encourage the search for these factors in health examination and stimulate the translational research on ILD development.

## Abbreviation

CI: Confidence interval
COPD: Chronic obstructive pulmonary disease
GERD: Gastroesophageal reflux disorder
HR: Hazard ratio
ICD: International classification of disease
ILD: Interstitial lung disease
IPF: Idiopathic pulmonary fibrosis
NHI: National health insurance
